# Rye Affects Bacterial Translocation, Intestinal Viscosity, Microbiota Composition and Bone Mineralization in Turkey Poults

**DOI:** 10.1371/journal.pone.0122390

**Published:** 2015-04-07

**Authors:** Guillermo Tellez, Juan D. Latorre, Vivek A. Kuttappan, Billy M. Hargis, Xochitl Hernandez-Velasco

**Affiliations:** 1 Department of Poultry Science, University of Arkansas, Fayetteville, Arkansas, United States of America; 2 Facultad de Medicina Veterinaria y Zootecnia, Universidad Nacional Autónoma de México, México D.F., México; Colorado State University, UNITED STATES

## Abstract

Previously, we have reported that rye significantly increased both viscosity and *Clostridium perfringens* proliferation when compared with corn in an *in vitro* digestive model. Two independent trials were conducted to evaluate the effect of rye as a source of energy on bacterial translocation, intestinal viscosity, gut microbiota composition, and bone mineralization, when compared with corn in turkey poults. In each experiment, day-of-hatch, turkey poults were randomly assigned to either a corn or a rye diet (*n* = 0 /group). At 10 d of age, in both experiments, 12 birds/group were given an oral gavage dose of fluorescein isothiocyanate dextran (FITC-d). After 2.5 h of oral gavage, blood and liver samples were collected to evaluate the passage of FITC-d and bacterial translocation (BT) respectively. Duodenum, ileum and cecum gut sections were collected to evaluate intestinal viscosity and to enumerate gut microbiota. Tibias were collected for observation of bone parameters. Broilers fed with a rye diet showed increased (*p*<0.05) intestinal viscosity, BT, and serum FITC-d. Bacterial enumeration revealed that turkey poults fed with rye had increased the number of total lactic acid bacteria (LAB) in all three sections of the gastrointestinal tract evaluated when compared to turkey poults fed with corn. Turkey poults fed with rye also had significantly higher coliforms in duodenum and ileum but not in the ceca, whereas the total number of anaerobes increased only in duodenum. A significant reduction in bone strength and bone mineralization was observed in turkey poults fed with rye when compared with corn fed turkey poults. In conclusion, rye evoked mucosal damage in turkey poults that increased intestinal viscosity, increased leakage through the intestinal tract, and altered the microbiota composition and bone mineralization. Studies to evaluate dietary inclusion of selected Direct-Fed Microbial (DFM) candidates that produce exogenous enzymes in rye fed turkey poults are currently being evaluated.

## Introduction

The intestinal epithelium constitutes the largest and most important barrier against external environmental agents and has three critical functions: 1) To prevent the entry of harmful intraluminal microorganisms, antigens, and toxins; 2) To enable the selective translocation of dietary nutrients and electrolytes into circulation; and 3) To tolerate the beneficial microbiome [1–4]. Inappropriate immunological reactions against food compounds, such as lactose or gluten, can lead to the breakdown of oral tolerance and the development of intestinal immune disorders in humans [5–16] and several investigators have described how the composition of the diet, also has a tremendous impact in digestibility and gut health of [17–19]. Thousands of years of evolution shaped the digestive system of the jungle fowl and wild pig to deal with the dietary ingredients they encounter in an efficient manner. More recently, throw intensive genetic manipulation, nutrition and health programs we have modified the biology and growth potential of production animals. Today, modem commercial monogastric animals diets contain 2 or 3 ingredients that may constitute >75% of intake. Corn is usually the main source of energy in poultry diets, but at times it is difficult to formulate least cost diets using corn, therefore, unconventional grains have to be used. Specifically, rye-based diets *versus* traditional corn-based diets, were different cereals are used as principal source of energy. The inclusion of rye in poultry diets has been fraught with problems, principally related to the production of sticky droppings, malabsorption syndrome, elevated feed conversion and intestinal bacterial overgrowth [20–22]. The endosperm cell wall of rye and wheat is comprised mainly of highly branched arabinoxylans which increase the viscosity of the digesta [23]. Elevated viscosity reduces digestibility and performance by interfering with the movement of particles and solutes across the intestinal lumen [24] favoring intestinal bacterial overgrowth [22]. The purpose of the present study was to evaluate the use of rye on bacterial translocation (BT), intestinal viscosity, microbiota composition, and bone mineralization when compared with a traditional cereal (corn) in turkey poults.

## Material and Methods

### Animal source, diets and experimental design

In order to show that the same or similar results can be achieved independently, two independent experiments were conducted in the present study. In each experiment, forty turkey poults were obtained from a commercial hatchery (Cargill Gentry, AR, USA), randomly assigned to 2 groups (*n* = 20 turkey poults), and placed into isolator chambers in a controlled age-appropriate environment with unrestricted access to feed and water for 10 days. Turkey poults received either a corn or rye diet meeting the nutritional requirements of poultry recommended by National Research Council [25]. Diets were antibiotic-free (Table 1). All animal handling procedures were in compliance with Institutional Animal Care and Use Committee (IACUC) at the University of Arkansas. Specifically, the IACUC approved this study under the protocol #11047- “Evaluation of direct fed microbials and prebiotics in poultry”. At ten days of age, in both experiments, 12 turkey poults in both treatment groups were randomly selected, and given an oral gavage dose of fluorescein isothiocyanate dextran (FITC-d; 2.2mg/mL/bird; MW 3,000–5,000 Da; Sigma Aldrich Co., St. Louis, MO). After 2.5 h they were humanly killed using carbon dioxide asphyxiation method. Blood samples were collected from the femoral vein for measuring leakage of FITC-d. Liver was collected to evaluate BT. Duodenal, ileal, and cecum gut sections were collected to enumerate bacteria. For intestinal viscosity, 5 turkey poults from each group ere humanly killed and intestinal digesta were individually collected. Additionally, tibias were collected for bone parameters as describe below.

**Table 1:** Composition of the experimental diets (g/kg).

**Diet**	**Maize-based diet**	**Rye-based diet**
**Ingredients**		
Maize (82.7 g/kg CP)	377.4	0.0
Rye (126 g/kg CP)	0.0	372.4
Soybean meal (474.2 g/kg CP)	517.3	482.2
Poultry oil	40.0	79.5
Dicalcium phosphate	36.8	36.6
Ground limestone	11.6	11.3
Sodium chloride	4.1	4.1
DL-Methionine	4.0	4.3
Vitamin premix^1^	1.0	1.0
L-Lysine HCl	4.5	5.0
Choline chloride 60%	1.0	1.0
Mineral premix^2^	1.0	1.0
Threonine	0.9	1.2
Selenium	0.2	0.2
Antioxidant^3^	0.2	0.2
**Calculated analysis**		
ME, MJ/kg	11.9	11.9
Crude protein, g/kg	285	285
Crude fat, g/kg	62.8	96.2
Calcium, g/kg	14.9	14.9
Total phosphorus, g/kg	10.9	10.9
Lysine, g/kg	18.2	18.2
Methionine, g/kg	7.7	7.9
Methionine + cysteine, g/kg	11.8	11.8

^1^Vitamin premix supplied per kg of diet: Retinol, 9.2 mg; cholecalciferol, 100 μg; dl-α-tocopherol, 90 mg; menadione, 6 mg; thiamine, 6.2 mg; riboflavin, 26.5 mg; pantothenic acid, 39.7 mg; niacin, 100 mg; pyridoxine, 11 mg; folic acid, 4 mg; biotin, 0.3 mg; cyanocobalamin, 0.1 mg.

^2^Mineral premix supplied per kg of diet: Mn, 70 mg; Zn, 40 mg; Fe, 37 mg; Cu, 6 mg; I, 0.7mg; Co, 0.2 mg.

^3^Ethoxyquin.

### Viscosity

Total intestinal digesta contents were collected from Meckel's diverticulum to the ileocecocolonic junction. For viscosity analysis, approximately 1.5 g (wet weight) of the fresh digesta was immediately placed in a microcentrifuge tube and centrifuged at 12,000 x *g* at 4°C for 5 min. The supernatant fluid was collected and stored on ice until viscosity measurement was determined using a LVDV-I Brookfield digital cone-plate viscometer fitted with a CP-40 spindle (Brookfield Engineering, Middleboro, MA). The analyzed samples and the viscometer cup were maintained at 40°C during viscosity measurement. Viscosity was measured in centipoise (cP = 1/100 dyne s/cm^2^) and the results were reported as log_10_ cP.

### Bacterial translocation

Briefly, the right half of the liver was removed from each turkey poults, collected in sterile bags, homogenized, weighed and 1:4 wt/vol dilutions were made with sterile 0.9% saline. Ten-fold dilutions of each sample, from each group were made in a sterile 96 well Bacti flat bottom plate and the diluted samples were plated on MacConkey Agar (VWR Cat. No. 89429–342 Suwanee, GA 30024) to evaluate total counts of *Enterobacteriaceae* per gram of tissue as has been previously described [11,26,27].

### Serum determination of FITC-d

Blood was kept at room temperature for 3 h and centrifuged (1,000 x *g* for 15 min) to separate the serum from the red blood cells. FITC-d levels of undiluted serum were measured at excitation wavelength of 485nm and emission wavelength of 528nm (Synergy HT, Multi-mode microplate reader, BioTek Instruments, Inc., Vermont, USA). Fluorescence measured was then compared to a standard curve with known FITC-d concentrations. Gut leakage for each bird was reported as μg of FITC-d/mL of serum.

### Enumeration of bacteria

Whole duodenum, ileum, and both ceca were aseptically removed, separated into sterile bags, and homogenized. Samples were weighed and 1:4 wt/vol dilutions were made with sterile 0.9% saline. Ten-fold dilutions of each sample, from each group were made in a sterile 96 well Bacti flat bottom plate and the diluted samples were plated on three different culture media; to evaluate total number of lactic acid bacteria (LAB) in Man Rogosa Sharpe (Difco Lactobacilli MRS Agar VWR Cat. No. 90004–084 Suwanee, GA 30024); total *Enterobacteriaceae* in MacConkey; and total anaerobes in tryptic soy agar with sodium thioglycolate plates (TSA, catalog no. 211822, Becton Dickinson, Sparks, MD).

### Bone parameters

Bone parameters were measured according to the methods as described by Zhang and Coon, [28]. Tibias from each turkey poults were cleaned of attached tissues. Bones from the left leg were subjected to conventional bone assays and tibia from the right leg was used to determine breaking strength. The bones from left tibia were dried at 100°C for 24 h and weighed again. The samples were then incinerated in a muffle furnace (Isotemp muffle furnace, Fisher Scientific, Pittsburgh, PA) at 600°C for 24 h in crucibles. Finally, the content of calcium and phosphorus in the tibia was determined using standard methods [29] and were reported as percentage of dry matter. The right tibial diaphyses from individual birds were cleaned of adherent tissues, the periosteum was removed, and the biomechanical strength of each bone was measured using an Instron 4502 (Norwood, MA) material testing machine with a 100 kg Load Cell. The bones were held in identical positions and the mid-diaphyseal diameter of the bone at the site of impact was measured using a dial caliper. The maximum load at failure was determined using a three-point flexural bend fixture with a total distance of 30 mm between the two lower supporting ends. The load, defined as force in kilograms per square millimeter of cross-sectional area (kg/mm^2^), represents bone strength. The rate of loading was kept constant at 20 mm/min collecting 10 data points per second. The data were automatically calculated using Instron’s Series IX Software (Norwood, MA).

### Statistical analysis

All data were subjected to one-way analysis of variance as a completely randomized design using the General Linear Models procedure of SAS [30]. Data are expressed as mean ± standard error. Significant differences among the means were determined by using Duncan’s multiple-range test at *p*<0.05.

## Results

The evaluation of body weight, intestinal viscosity, serum FITC-d, and liver BT in turkey poults fed with a corn diet or a rye diet of Experiment 1 and 2 are summarized in Table 2. A significant (*p*<0.05) reduction in body weight was observed in turkey poults fed with rye as compared with corn in both experiments. However, turkey poults fed with rye showed an increase in intestinal viscosity which was associated with elevated (*p*<0.05) serum FITC-d, and an increase in BT of *Enterobacteriaceae* to the liver (Table 2).

**Table 2:** Evaluation of body weight, intestinal viscosity, serum FITC-d, and liver bacterial translocation in turkey poults fed with corn or rye in Experiment 1 and 2.

**Group**	**Body weight (g)**	**Intestinal viscosity(cP Log**_10_ **)**	**Serum FITC-d (μg/mL)**	**Bacterial translocation of** *Enterobacteriaceae* **(CFU Log**_10_ **)**
**Experiment 1**				
**Corn**	133.64 ± 3.49^a^	0.20 ± 0.07^b^	0.23 ± 0.01^b^	2.13 ± 0.43^b^
**Rye**	75.91 ± 3.61^b^	2.99 ± 0.97^a^	0.53 ± 0.05^a^	4.03 ± 0.51^a^
**Experiment 2**				
**Corn**	111.56 ± 1.54^a^	0.25 ± 0.64^b^	0.51 ± 0.13^b^	1.98 ± 0.891^b^
**Rye**	76.47 ± 1.59^b^	3.30 ± 0.87^a^	0.72 ± 0.17^a^	3.88 ± 0.43^a^

^a-b^Superscripts within columns indicate significant difference at *p*<0.05.

Data is expressed as Mean ± SE.

Intestinal viscosity is expressed in Log_10_ (in centipoise, cP = 1/100 dyne sec/cm^2^) from 5 turkey poults.

Serum FITC-d and liver bacterial translocation (expressed in cfu Log_10_/g of tissue) from 12 turkey poults.

Total bacterial counts in duodenum, ileum, and ceca of neonatal turkey poults fed with a corn or rye diet in Experiment 1 and 2 are summarized in Table 3. In both trials, turkey poults that were fed with rye had a significant increase in the number of total LAB that were observed in duodenum, in ileum, and ceca when compared with turkey poults fed with corn. In these turkey poults, a significant increase in the total number of coliforms was also observed in duodenum and ileum but not in cecum, whereas, an increase in total number of anaerobes was observed only in the duodenum (Table 3).

**Table 3:** Evaluation of total bacterial counts in duodenum, ileum, or ceca in turkey poults fed with corn or rye in Experiments 1 and 2.

**Group**	**Duodenum**			**Ileum**				**Ceca**		
	**Coliforms**	**LAB’s**	**Anaerobes**	**Coliforms**	**LAB’s**	**Anaerobes**		**Coliforms**	**LAB’s**	**Anaerobes**
**Experiment 1**										
**Corn**	1.0 ± 0.19^b^	2.29 ± 0.76^b^	3.86 ± 0.40^b^	1.92 ± 0.11^b^	3.98± 0.58^b^	5.16 ± 0.11^a^		7.75 ± 0.14^a^	7.02 ± 0.14^b^	7.91 ± 0.20^a^
**Rye**	3.62 ± 0.35^a^	5.91 ± 0.25^a^	5.36 ± 0.31^a^	4.87 ± 0.70^a^	6.25 ± 0.57^a^	5.41 ± 0.50^a^		7.48 ± 0.15^a^	8.02 ± 0.14^a^	7.83 ± 0.30^a^
**Experiment 2**										
**Corn**	2.27 ± 0^b^	3.27 ± 0.76^b^	3.27 ± 0.40^b^	1.42 ± 0.11^b^	3.42± 0.58^b^	3.42 ± 0.11^a^	8.08 ± 0.14^a^		7.08 ± 0.14^b^	8.08 ± 0.20^a^
**Rye**	3.77 ± 0.35^a^	5.77 ± 0.25^a^	6.23 ± 0.31^a^	3.66 ± 0.70^a^	6.66 ± 0.57^a^	3.66 ± 0.50^a^	7.71 ± 0.15^a^		7.71 ± 0.14^a^	7.71 ± 0.30^a^

^a-b^Superscripts within columns indicate significant difference at *p*<0.05

Data is expressed in Log_10_ cfu /g of tissue. Mean ± SE from 12 turkey poults.

The results of the evaluation of bone breaking strength and bone parameters in neonatal turkey poults fed with corn or rye in Experiments 1 and 2 are summarized in Table 4. Significant increases in tibia diameter, tibia breaking strength, tibia ash, and calcium and phosphorus percentages were observed in turkey poults that fed corn when compared with turkey poults that fed the rye diet (Table 4).

**Table 4:** Evaluation of bone breaking strength and bone parameters in turkey poults fed with corn or rye in Experiments 1 and 2.

**Groups**	**Tibia strength Load at yield (kg)**	**Tibia diameter (mm)**	**Breaking strength (kg/mm** ^2^ **)**	**Total ash from tibia (%)**	**Calcium (% of ash)**	**Phosphorus(% of ash)**
**Experiment 1**						
**Corn**	5.04 ± 0.011 ^a^	3.34 ± 0.17 ^a^	1.59 ± 0.04 ^a^	55.01 ± 0.41 ^a^	29.48 ± 0.27 ^a^	18.15 ± 0.12 ^a^
**Rye**	1.58 ± 0.009 ^b^	1.61 ± 0.28 ^b^	0.90 ± 0.02 ^b^	34.87 ± 0.35 ^b^	18.48 ± 0.27 ^b^	13.15 ± 0.12 ^b^
**Experiment 2**						
**Corn**	6.14 ± 0.01 ^a^	4.55 ± 0.32 ^a^	1.90 ± 0.03 ^a^	65.61 ± 0.81 ^a^	37.65 ± 0.07 ^a^	21.35 ± 0.52 ^a^
**Rye**	2.08 ± 0.03 ^b^	1.82 ± 0.78 ^b^	0.99 ± 0.02 ^b^	30.87 ± 0.75 ^b^	21.32 ± 0.46 ^b^	15.67 ± 0.29 ^b^

Tibias from twelve chickens were collected to evaluate bone qualities. Data is expressed as mean ± standard error.

^a-b^Superscripts within columns indicate significant difference at *p*< 0.05 Values within a row with no common superscript differ significantly *p*<0.05.

## Discussion

Feeding cereals high in non-starch polysaccharides (NSPs) leads to increased feed conversion ratios and lower body-weight gain [31–33]. In addition, high NSPs diets have also been associated with Necrotic Enteritis (NE), a multi-factorial disease caused by *Clostridium perfringens* that is probably the most important bacterial disease in terms of economic implications in turkey poults [24,34,35]. Since poultry has little or no intrinsic enzymes capable of hydrolyzing these NSPs, exogenous xylanases as additives are used in an attempt to reduce this anti-nutritive factors [21,23,36]. Several mechanisms of action of NSPs on nutrient absorption have been described that include an increased digesta viscosity, thickening of the mucous layer on the intestinal mucosa, epithelial cell apoptosis, inflammation in response to the dysbacteriosis caused by a higher soluble NSPs content [30,37–39], and the nutritional and economic consequences of mounting an inflammatory response in poultry is inversely related to body-weight gain and overall performance [22,40]. In the present study, turkey poults fed with rye showed an increase in intestinal viscosity, elevated BT of *Enterobacteriaceae*, and increased serum FITC-d (Table 2). These changes were also associated with significant bacterial overgrowth when compared with turkey poults fed with corn (Table 3). It is important to mention that the total bacterial counts were evaluated on selective media and using standard microbiological procedures that are known to severely under-represent the true microbiological diversity. Nevertheless, it provides interesting results that justify further exploration using metagenomics analysis. Variations in the composition of the microbiome within different segments of the alimentary tract are influenced by the environment, by the diet, and by the host [41–44]. Alterations in gut permeability are associated with BT in the portal and/or systemic circulation in several types of leaky gut syndromes leading to systemic bacterial infections [26,45]. Similarly, FITC-d is a large molecule (3–5 kDa) which does not usually leak through the intact gastrointestinal tract barrier. However, when conditions disrupt the tight junctions between epithelial cells, the FITC-d molecule can enter circulation as demonstrated by an increase in trans-mucosal permeability associated with chemically induced disruption of tight junctions by elevated serum levels of FITC-d after oral administration [46]. The significant reduction in bone strength and mineralization (Table 4) confirmed previous studies that have shown that high NSPs diets in poultry or gluten intolerance in humans, are also associated with malabsorption of minerals and fat-soluble vitamins [13,47–51]. Performance of rye-fed birds can be improved markedly by dietary supplementation with exogenous xylanase [21,52,53]. Previously, we have reported that dietary inclusion of selected Direct-Fed Microbial (DFM) candidates that produce exogenous enzymes (protease, phytase, lipase, xylanase and cellulose) in high NSPs diets significantly reduced both viscosity and *Clostridium perfringens* proliferation when compared with control diets without the DFM *in vitro* [27,54,55]. Together, they represent a step towards the application of nutrigenomics in the context of a chicken model. The incorporation of one or more nutrigenomics techniques (in particular, assessment of the microbiome) will provide a better understanding of how dietary food components can affect physiological functions and the fundamental cellular and molecular mechanisms implicated in the digestive process of high NSPs diets in chickens.

In conclusion, rye evoked mucosal damage in turkey poults resulting in increased intestinal viscosity, increased leakage through disruption of epithelial tight junctions in the intestinal tract, and altered the microbiota composition and bone mineralization. Studies to evaluate dietary inclusion of selected DFM candidates that produce exogenous enzymes in rye fed turkey poults are currently being evaluated.

## Supporting Information

S1 Dataset Dataset trial 1 and 2. (XLSX)
